# Supplementary material for: Genetic and Evolutionary Analysis of Porcine Deltacoronavirus in Guangxi Province, Southern China, from 2020 to 2023
Source: Microorganisms. 2024 Feb 19;12(2):416. doi: 10.3390/microorganisms12020416 (PMC10893222; doi:10.3390/microorganisms12020416)
Supplement: Supplementary file 1 [file microorganisms-12-00416-s001.zip › microorganisms-2854249-supplementary.pdf]

Supplementary

Supplementary Table S1. The information of S gene of PDCoV strains used in this study.

Virus strain	Date	Origin	Accession No.	Virus strain	Date	Origin	Accession No.
CHAH-KP757890-2004	2004	Anhui, CHN	KP757890	USA-KR265847-2014	2014	USA	KR265847
CHHK-NC039208-2010	2010	Hong Kong, CHN	NC039208	USA-KR265848-2014	2014	USA	KR265848
CHHK-JQ065043-2010	2010	Hong Kong, CHN	JQ065043	USA-KR265849-2014	2014	USA	KR265849
CHHK-JQ065042-2009	2009	Hong Kong, CHN	JQ065042	USA-KR265850-2014	2014	USA	KR265850
CHSD-MF431743-2014	2014	Shandong, CHN	MF431743	USA-KR265851-2014	2014	USA	KR265851
CHHN-KT336560-2014	2014	Henan, CHN	KT336560	USA-KR265852-2014	2014	USA	KR265852
CHHuN-KY513724-2014	2014	Hunan, CHN	KY513724	USA-KR265853-2014	2014	USA	KR265853
CHHuB-KP757891-2014	2014	Hubei, CHN	KP757891	USA-KR265854-2014	2014	USA	KR265854
CHJS-KP757892-2014	2014	Jiangsu, CHN	KP757892	USA-KR584357-2014	2014	USA	KR584357
CHJS-KU665558-2014	2014	Jiangsu, CHN	KU665558	USA-KR584358-2014	2014	USA	KR584358
CHHeN-MN942260-2015	2015	Henan, CHN	MN942260	USA-KR584359-2014	2014	USA	KR584359
CHJX-KR131621-2015	2015	Jiangxi, CHN	KR131621	USA-KR584360-2014	2014	USA	KR584360
CHGD-MF431742-2015	2015	Guangdong, CHN	MF431742	USA-KR584361-2014	2014	USA	KR584361
CHSXD-KT021234-2015	2015	Shaanxi, CHN	KT021234	USA-KR584362-2014	2014	USA	KR584362
CHTW-MW853634-2015	2015	Taiwan, CHN	MW853634	USA-KR584363-2014	2014	USA	KR584363
CHGX-KU204697-2015	2015	Guangxi, GX, CHN	KU204697	USA-KR584364-2014	2014	USA	KR584364
CHHN-KX443143-2016	2016	Henan, CHN	KX443143	USA-KR584365-2014	2014	USA	KR584365
CHHN-MG832584-2016	2016	Henan, CHN	MG832584	USA-KJ584355-2014	2014	USA	KJ584355
CHGS-MF642322-2016	2016	Gansu, CHN	MF642322	USA-KJ584356-2014	2014	USA	KJ584356
CHGS-MF642323-2016	2016	Gansu, CHN	MF642323	USA-KJ584357-2014	2014	USA	KJ584357
CHGS-MF642324-2016	2016	Gansu, CHN	MF642324	USA-KJ584358-2014	2014	USA	KJ584358
CHGS-MF642325-2016	2016	Gansu, CHN	MF642325	USA-KJ584359-2014	2014	USA	KJ584359

CHSC-MK355396-2016	2016	Sichuan, CHN	MK355396	USA-KJ620016-2014	2014	USA	KJ620016
CHJX-KY293677-2016	2016	Jianxi, CHN	KY293677	USA-MZ291567-2014	2014	USA	MZ291567
CHJX-KY293678-2016	2016	Jianxi, CHN	KY293678	USA-KJ362462-2014	2014	USA	KJ362462
CHGD-MH715491-2016	2016	Guangdong, CHN	MH715491	USA-KM012168-2014	2014	USA	KM012168
CHGD-KY363868-2016	2016	Guangdong, CHN	KY363868	USA-KJ569769-2014	2014	USA	KJ569769
CHTJ-KY065120-2016	2016	Tianjin, CHN	KY065120	USA-KJ584352-2014	2014	USA	KJ584352
CHSH-MF041982-2016	2016	Shanghai, CHN	MF041982	USA-KJ481931-2014	2014	USA	KJ481931
CHGX-MZ388469-2016	2016	Guangxi, GX, CHN	MZ388469	USA-KJ601779-2014	2014	USA	KJ601779
CHHB-MF948005-2017	2017	Hebei, CHN	MF948005	USA-KJ567050-2014	2014	USA	KJ567050
CHHB-MF037204-2017	2017	Hebei, CHN	MF037204	USA-KT381613-2014	2014	USA	KT381613
CHHeB-MG242062-2017	2017	Hebei, CHN	MG242062	USA-KX022602-2015	2015	USA	KX022602
CHSC-MK330604-2017	2017	Sichuan, CHN	MK330604	USA-KX022603-2015	2015	USA	KX022603
CHSC-MK572803-2017	2017	Sichuan, CHN	MK572803	USA-KX022604-2015	2015	USA	KX022604
CHSC-MK211169-2017	2017	Sichuan, CHN	MK211169	USA-KX022605-2015	2015	USA	KX022605
CHHG-MF095123-2017	2017	Hubei, CHN	MF095123	USA-KR150443-2015	2015	USA	KR150443
CHJS-MN249445-2017	2017	Jiangsu, CHN	MN249445	JPN-LC260038-2014	2014	Japan	LC260038
CHJS-MN249445-2017	2017	Jiangsu, CHN	MN249445	JPN-LC260039-2014	2014	Japan	LC260039
CHHK-MT263013-2017	2017	Hong Kong, CHN	MT263013	JPN-LC260040-2014	2014	Japan	LC260040
CHGX-MZ388470-2017	2017	Guangxi, CHN	MZ388470	JPN-LC260042-2014	2014	Japan	LC260042
CHGX-MZ388471-2017	2017	Guangxi, CHN	MZ388471	JPN-LC260043-2014	2014	Japan	LC260043
CHGX-MZ388472-2017	2017	Guangxi, CHN	MZ388472	JPN-LC260045-2014	2014	Japan	LC260045
CHGX-MT505445-2017	2017	Guangxi, CHN	MT505445	KOR-KM820765-2014	2014	Korea	KM820765
CHGX-MT505446-2017	2017	Guangxi, CHN	MT505446	KOR-MG837130-2016	2016	Korea	MG837130
CHGX-MT505447-2017	2017	Guangxi, CHN	MT505447	KOR-MG837131-2016	2016	Korea	MG837131
CHGX-MT505448-2017	2017	Guangxi, CHN	MT505448	KOR-MG837132-2016	2016	Korea	MG837132
CHGX-MT505449-2017	2017	Guangxi, CHN	MT505449	KOR-MG837133-2016	2016	Korea	MG837133
CHGX-MK040449-2017	2017	Guangxi, CHN	MK040449	KOR-KY354363-2016	2016	Korea	KY354363

CHGX-MK040450-2017	2017	Guangxi, CHN	MK040450	KOR-KY354564-2016	2016	Korea	KY354564
CHGX-MN025260-2017	2017	Guangxi, CHN	MN025260	KOR-KY926512-2016	2016	Korea	KY926512
CHJX-MK625638-2018	2018	Jianxi, CHN	MK625638	Haiti-MW685623-2015	2015	Haiti	MW685623
CHJX-MK625639-2018	2018	Jianxi, CHN	MK625639	MEXICO-MK478383-2017	2017	Mexico	MK478383
CHJX-MK625640-2018	2018	Jianxi, CHN	MK625640	Peru-MT227371-2019	2019	Peru	MT227371
CHJX-MK625641-2018	2018	Jianxi, CHN	MK625641	Thailand-KX361343-2013	2013	Thailand	KX361343
CHSC-MK330605-2018	2018	Sichuan, CHN	MK330605	Thailand-KX361344-2013	2013	Thailand	KX361344
CHSC-MK005882-2018	2018	Sichuan, CHN	MK005882	Thailand-KX361345-2015	2015	Thailand	KX361345
CHSD-MN173809-2018	2018	Shandong, CHN	MN173809	Thailand-KU051641-2015	2015	Thailand	KU051641
CHSD-MN173810-2018	2018	Shandong, CHN	MN173810	Thailand-KU051649-2015	2015	Thailand	KU051649
CHSD-MN173811-2018	2018	Shandong, CHN	MN173811	Thailand-MZ802772-2015	2015	Thailand	MZ802772
CHSD-MN173812-2018	2018	Shandong, CHN	MN173812	Thailand-MZ802775-2015	2015	Thailand	MZ802775
CHSD-MN173803-2018	2018	Shandong, CHN	MN173803	Thailand-KU984334-2015	2015	Thailand	KU984334
CHSD-MN173805-2018	2018	Shandong, CHN	MN173805	Thailand-MZ802773-2016	2016	Thailand	MZ802773
CHSD-MN173806-2018	2018	Shandong, CHN	MN173806	Thailand-MZ802774-2016	2016	Thailand	MZ802774
CHSD-MN173807-2018	2018	Shandong, CHN	MN173807	Thailand-MZ802777-2016	2016	Thailand	MZ802777
CHHN-MH708123-2018	2018	Henan, CHN	MH708123	Vietnam-KX834351-2015	2015	Vietnam	KX834351
CHHN-MH708124-2018	2018	Henan, CHN	MH708124	Vietnam-KX834352-2015	2015	Vietnam	KX834352
CHHN-MT260149-2018	2018	Henan, CHN	MT260149	Vietnam-KX998969-2015	2015	Vietnam	KX998969
CHGX-MZ388473-2018	2018	Guangxi, CHN	MZ388473	Vietnam-MH118332-2015	2015	Vietnam	MH118332
CHGX-MZ388474-2018	2018	Guangxi, CHN	MZ388474	Vietnam-MZ802776-2016	2016	Vietnam	MZ802776
CHGX-MK359104-2018	2018	Guangxi, CHN	MK359104	CHGX-BS01-2020	2020	Baise, GX, CHN	OR659117
CHGX-MN173782-2018	2018	Guangxi, CHN	MN173782	CHGX-BS02-2020	2020	Baise, GX, CHN	OR659118
CHGX-MN173779-2018	2018	Guangxi, CHN	MN173779	CHGX-BS03-2020	2020	Baise, GX, CHN	OR659119
CHGX-MN173780-2018	2018	Guangxi, CHN	MN173780	CHGX-BS01-2021	2021	Baise, GX, CHN	OR659120
CHGX-MN173781-2018	2018	Guangxi, CHN	MN173781	CHGX-BS02-2021	2021	Baise, GX, CHN	OR659121
CHGX-MT505450-2018	2018	Guangxi, CHN	MT505450	CHGX-CZ01-2021	2021	Chongzuo, GX, CHN	OR659136

CHGX-MT505451-2018	2018	Guangxi, CHN	MT505451	CHGX-CZ02-2021	2021	Chongzuo, GX, CHN	OR659137
CHGX-MT505452-2018	2018	Guangxi, CHN	MT505452	CHGX-CZ03-2021	2021	Chongzuo, GX, CHN	OR659138
CHGX-MT505453-2018	2018	Guangxi, CHN	MT505453	CHGX-CZ04-2021	2021	Chongzuo, GX, CHN	OR659139
CHGX-MT505454-2018	2018	Guangxi, CHN	MT505454	CHGX-GG01-2022	2022	Guigang, GX, CHN	OR659140
CHGX-MT505455-2018	2018	Guangxi, CHN	MT505455	CHGX-GG02-2022	2022	Guigang, GX, CHN	OR659141
CHGX-MN520203-2018	2018	Guangxi, CHN	MN520203	CHGX-GG03-2022	2022	Guigang, GX, CHN	OR659142
CHGX-MN058073-2018	2018	Guangxi, CHN	MN058073	CHGX-GG04-2022	2022	Guigang, GX, CHN	OR659143
CHAN-MN520198-2019	2019	Anhui, CHN	MN520198	CHGX-GG05-2022	2022	Guigang, GX, CHN	OR659144
CHTS-MT663769-2019	2019	Hebei, CHN	MT663769	CHGX-GG06-2022	2022	Guigang, GX, CHN	OR659145
CHSC-MK993519-2019	2019	Sichuan, CHN	MK993519	CHGX-GG07-2022	2022	Guigang, GX, CHN	OR659146
CHSD-MN520191-2019	2019	Shandong, CHN	MN520191	CHGX-NN01-2021	2021	Nanning, GX, CHN	OR659147
CHGX-MT505456-2019	2019	Guangxi, CHN	MT505456	CHGX-NN02-2021	2021	Nanning, GX, CHN	OR659148
CHGX-MT505457-2019	2019	Guangxi, CHN	MT505457	CHGX-NN03-2021	2021	Nanning, GX, CHN	OR659149
CHGX-MT505458-2019	2019	Guangxi, CHN	MT505458	CHGX-BS01-2022	2022	Baise, GX, CHN	OR659122
CHGX-MT505459-2019	2019	Guangxi, CHN	MT505459	CHGX-BS02-2022	2022	Baise, GX, CHN	OR659123
CHGX-MT505460-2019	2019	Guangxi, CHN	MT505460	CHGX-BS03-2022	2022	Baise, GX, CHN	OR659124
CHHLJ-MZ802955-2020	2020	Heilongjiang, CHN	MZ802955	CHGX-BS04-2022	2022	Baise, GX, CHN	OR659125
CHGX-OL441343-2020	2020	Guangxi, CHN	OL441343	CHGX-BS05-2022	2022	Baise, GX, CHN	OR659126
CHTW-MZ712033-2021	2021	Taiwan, CHN	MZ712033	CHGX-BS06-2022	2022	Baise, GX, CHN	OR659127
CHTW-MZ712034-2021	2021	Taiwan, CHN	MZ712034	CHGX-BS07-2022	2022	Baise, GX, CHN	OR659128
CHTW-MZ712035-2021	2021	Taiwan, CHN	MZ712035	CHGX-BS08-2022	2022	Baise, GX, CHN	OR659129
CHTW-MZ712036-2021	2021	Taiwan, CHN	MZ712036	CHGX-BS09-2022	2022	Baise, GX, CHN	OR659130
CHTW-MZ712037-2021	2021	Taiwan, CHN	MZ712037	CHGX-BS10-2022	2022	Baise, GX, CHN	OR659131
CHTW-MZ712038-2021	2021	Taiwan, CHN	MZ712038	CHGX-BS11-2022	2022	Baise, GX, CHN	OR659132
CHTW-MZ712039-2021	2021	Taiwan, CHN	MZ712039	CHGX-BS12-2022	2022	Baise, GX, CHN	OR659133
CHTW-MZ712040-2021	2021	Taiwan, CHN	MZ712040	CHGX-BS13-2022	2022	Baise, GX, CHN	OR659134
CHTW-OM777140-2021	2021	Taiwan, CHN	OM777140	CHGX-BS14-2022	2022	Baise, GX, CHN	OR659135

CHTW-OL840383-2021	2021	Taiwan,CHN	OL840383	CHGX-YL01-2023	2023	Yulin, GX, CHN	OR659150
CHSD-OM256446-2021	2021	Shandong, CHN	OM256446				
CHGX-OQ547740-2021	2021	Guangxi, CHN	OQ547740				
CHGX-OQ736717-2021	2021	Guangxi, CHN	OQ736717				
CHZJ-OR762053-2022	2022	Zhejiang,CHN	OR762053				
CHJS-OQ504186-2022	2022	Jiangsu,CHN	OQ504186				
CHGX-OQ736716-2022	2022	Guangxi, CHN	OQ736716				
CHHuN-OR269935-2023	2023	Hunan, CHN	OR269935				

Note: GX: Guangxi province of China; CHN: China; JPN: Japan; KOR: Korea; USA: the United States of America. The sequences obtained in this study are marked with red. The same as follows.

Supplementary Table S2. The information of M gene of PDCoV strains used in this study.

Virus strain	Date	Origin	Accession No.	Virus strain	Date	Origin	Accession No.
CHAN-KP757890-2004	2004	Anhui, CHN	KP757890	USA-KJ584355-2014	2014	USA	KJ584355
CHHK-JQ065042-2009	2009	Hong Kong, CHN	JQ065042	USA-KJ584356-2014	2014	USA	KJ584356
CHHK-JQ065043-2010	2010	Hong Kong, CHN	JQ065043	USA-KJ584357-2014	2014	USA	KJ584357
CHHK-NC039208-2010	2010	Hong Kong, CHN	NC039208	USA-KJ584358-2014	2014	USA	KJ584358
CHHuN-KY513724-2014	2014	Hunan, CHN	KY513724	USA-KJ584359-2014	2014	USA	KJ584359
CHHN-KT336560-2014	2014	Henan, CHN	KT336560	USA-KR265847-2014	2014	USA	KR265847
CHSD-MF431743-2014	2014	Shandong, CHN	MF431743	USA-KR265848-2014	2014	USA	KR265848
CHHB-KP757891-2014	2014	Hubei, CHN	KP757891	USA-KR265849-2014	2014	USA	KR265849
CHJS-KP757892-2014	2014	Jiangsu, CHN	KP757892	USA-KR265850-2014	2014	USA	KR265850
CHJS-KU665558-2014	2014	Jiangsu, CHN	KU665558	USA-KR265851-2014	2014	USA	KR265851
CHJS-KY513725-2014	2014	Jiangsu, CHN	KY513725	USA-KR265852-2014	2014	USA	KR265852
CHSD-MF431742-2015	2015	Shandong, CHN	MF431742	USA-KR265853-2013	2013	USA	KR265853
CHJX-KR131621-2015	2015	Jiangxi, CHN	KR131621	USA-KR265854-2014	2014	USA	KR265854
CHNH-KU981059-2015	2015	Heilongjiang, CHN	KU981059	USA-KR265855-2014	2014	USA	KR265855
CHSXD-KT021234-2015	2015	Shaanxi, CHN	KT021234	USA-KR265856-2014	2014	USA	KR265856
CHTW-MW854634-2015	2015	Taiwan, CHN	MW854634	USA-KR265857-2014	2014	USA	KR265857
CH-KX443143-2016	2016	Hennan, CHN	KX443143	USA-KR265858-2014	2014	USA	KR265858
CHGD-KY363868-2016	2016	Guangdong, CHN	KY363868	USA-KR265859-2014	2014	USA	KR265859
CHGS-MF642322-2016	2016	Gansu, CHN	MF642322	USA-KR265860-2014	2014	USA	KR265860
CHGS-MF642323-2016	2016	Gansu, CHN	MF642323	USA-KR265861-2014	2014	USA	KR265861
CHSC-MK355396-2016	2016	Sichuan, CHN	MK355396	USA-KR265862-2014	2014	USA	KR265862
CHHB-KY129985-2016	2016	Hubei, CHN	KY129985	USA-KR265863-2014	2014	USA	KR265863
CHJX-KY293677-2016	2016	Jiangxi, CHN	KY293677	USA-KR265864-2014	2014	USA	KR265864
CHJX-KY293678-2016	2016	Jiangxi, CHN	KY293678	USA-KR265865-2014	2014	USA	KR265865
CHGD-KY363867-2016	2016	Guangdong, CHN	KY363867	USA-KT381613-2014	2014	USA	KT381613

CHGD-MH715491-2016	2016	Guangdong, CHN	MH715491	USA-KJ567050-2014	2014	USA	KJ567050
CHGD-MF280390-2016	2016	Guangdong, CHN	MF280390	USA-KJ481931-2014	2014	USA	KJ481931
CHTJ-KY065120-2016	2016	Tianjin, CHN	KY065120	USA-KJ620016-2014	2014	USA	KJ620016
CHCQ-MZ772936-2016	2016	Chongqing, CHN	MZ772936	USA-KJ462462-2014	2014	USA	KJ462462
CHSH-MF041982-2016	2016	Shanghai, CHN	MF041982	USA-KJ569769-2014	2014	USA	KJ569769
CHGX-MZ388469-2016	2016	Guangxi, CHN	MZ388469	USA-KM012168-2014	2014	USA	KM012168
CHGS-MF642324-2017	2017	Gansu, CHN	MF642324	USA-MZ291567-2014	2014	USA	MZ291567
CHGS-MF642325-2017	2017	Gansu, CHN	MF642325	USA-KX022602-2015	2015	USA	KX022602
CHSC-MK330604-2017	2017	Sichuan, CHN	MK330604	USA-KX022604-2015	2015	USA	KX022604
CHSC-MK572803-2017	2017	Sichuan, CHN	MK572803	USA-KX022603-2015	2015	USA	KX022603
CHSC-MK211169-2017	2017	Sichuan, CHN	MK211169	USA-KX022605-2015	2015	USA	KX022605
CHHeB-MG242062-2017	2017	Hebei, CHN	MG242062	USA-KR150443-2015	2015	USA	KR150443
CHHB-MF948005-2017	2017	Hubei, CHN	MF948005	JPN-LC260038-2014	2014	JPN	LC260038
CHHG-MF572803-2017	2017	Hunan, CHN	MF572803	JPN-LC260039-2014	2014	JPN	LC260039
CHJS-MN249445-2017	2017	Jiangsu, CHN	MN249445	JPN-LC260040-2014	2014	JPN	LC260040
CHGX-MZ388470-2017	2017	Guangxi, CHN	MZ388470	JPN-LC260041-2014	2014	JPN	LC260041
CHGX-MZ388471-2017	2017	Guangxi, CHN	MZ388471	JPN-LC260042-2014	2014	JPN	LC260042
CHGX-MZ388472-2017	2017	Guangxi, CHN	MZ388472	JPN-LC260043-2014	2014	JPN	LC260043
CHGX-MT524746-2017	2017	Guangxi, CHN	MT524746	JPN-LC260044-2014	2014	JPN	LC260044
CHGX-MT524747-2017	2017	Guangxi, CHN	MT524747	JPN-LC260045-2016	2016	JPN	LC260045
CHGX-MT524748-2017	2017	Guangxi, CHN	MT524748	KOR-KM820765-2014	2014	KOR	KM820765
CHGX-MT524749-2017	2017	Guangxi, CHN	MT524749	KOR-KY364365-2014	2014	KOR	KY364365
CHGX-MT524750-2017	2017	Guangxi, CHN	MT524750	KOR-KY926512-2016	2016	KOR	KY926512
CHGX-MN025260-2017	2017	Guangxi, CHN	MN025260	KOR-KY354363-2016	2016	KOR	KY354363
CHSC-MK005882-2018	2018	Sichuan, CHN	MK005882	KOR-KY354364-2016	2016	KOR	KY354364
CHSC-MK330605-2018	2018	Sichuan, CHN	MK330605	KOR-MG837130-2016	2016	KOR	MG837130
CHHN-MH708123-2018	2018	Henan, CHN	MH708123	KOR-MG837131-2016	2016	KOR	MG837131

CHHN-MH708124-2018	2018	Henan, CHN	MH708124	KOR-MG837132-2016	2016	KOR	MG837132
CHHN-MH708125-2018	2018	Henan, CHN	MH708125	KOR-MG837133-2016	2016	KOR	MG837133
CHJX-MK625638-2018	2018	Jiangxi, CHN	MK625638	Thailand-KX613443-2013	2013	Thailand	KX613443
CHJX-MK625639-2018	2018	Jiangxi, CHN	MK625639	Thailand-KX361344-2013	2013	Thailand	KX361344
CHJX-MK625640-2018	2018	Jiangxi, CHN	MK625640	Thailand-KU984334-2015	2015	Thailand	KU984334
CHJX-MK625641-2018	2018	Jiangxi, CHN	MK625641	Thailand-KX361345-2015	2015	Thailand	KX361345
CHGX-MZ388473-2018	2018	Guangxi, CHN	MZ388473	Thailand-KU051641-2015	2015	Thailand	KU051641
CHGX-MZ388474-2018	2018	Guangxi, CHN	MZ388474	Thailand-KU051649-2015	2015	Thailand	KU051649
CHGX-MK359104-2018	2018	Guangxi, CHN	MK359104	Thailand-MZ802772-2016	2016	Thailand	MZ802772
CHGX-MN173782-2018	2018	Guangxi, CHN	MN173782	Thailand-MZ802773-2016	2016	Thailand	MZ802773
CHGX-MN173779-2018	2018	Guangxi, CHN	MN173779	Thailand-MZ802774-2016	2016	Thailand	MZ802774
CHGX-MN173780-2018	2018	Guangxi, CHN	MN173780	Thailand-MZ802775-2016	2016	Thailand	MZ802775
CHGX-MN173781-2018	2018	Guangxi, CHN	MN173781	Thailand-MZ802777-2016	2016	Thailand	MZ802777
CHGX-MT524751-2018	2018	Guangxi, CHN	MT524751	Vietnam-MH118332-2015	2015	Vietnam	MH118332
CHGX-MT524752-2018	2018	Guangxi, CHN	MT524752	Vietnam-MZ802776-2016	2016	Vietnam	MZ802776
CHGX-MT524753-2018	2018	Guangxi, CHN	MT524753	GXBS01-2020	2020	Baise, GX, CHN	OR659151
CHGX-MT524754-2018	2018	Guangxi, CHN	MT524754	GXBS02-2020	2020	Baise, GX, CHN	OR659152
CHGX-MT524755-2018	2018	Guangxi, CHN	MT524755	GXBS03-2020	2020	Baise, GX, CHN	OR659153
CHGX-MT524756-2018	2018	Guangxi, CHN	MT524756	GXBS01-2021	2021	Baise, GX, CHN	OR659154
CHGX-MN520203-2018	2018	Guangxi, CHN	MN520203	GXBS02-2021	2021	Baise, GX, CHN	OR659155
CHGX-MN058073-2018	2018	Guangxi, CHN	MN058073	GXCZ01-2021	2021	Chongzuo, GX, CHN	OR659170
CHTS-MT665769-2019	2019	Hebei, CHN	MT665769	GXCZ02-2021	2021	Chongzuo, GX, CHN	OR659171
CHSC-MK993519-2019	2019	Sichuan, CHN	MK993519	GXCZ03-2021	2021	Chongzuo, GX, CHN	OR659172
CHSD-MN520191-2019	2019	Shandong, CHN	MN520191	GXCZ04-2021	2021	Chongzuo, GX, CHN	OR659173
CHGX-MT524757-2019	2019	Guangxi, CHN	MT524757	GXNN01-2021	2021	Nanning, GX, CHN	OR659181
CHGX-MT524758-2019	2019	Guangxi, CHN	MT524758	GXNN02-2021	2021	Nanning, GX, CHN	OR659182
CHGX-MT524759-2019	2019	Guangxi, CHN	MT524759	GXNN03-2021	2021	Nanning, GX, CHN	OR659183

CHGX-MT524762-2019	2019	Guangxi, CHN	MT524762	GXBS01-2022	2022	Baise, GX, CHN	OR659156
CHGX-MT524761-2019	2019	Guangxi, CHN	MT524761	GXBS02-2022	2022	Baise, GX, CHN	OR659157
CHAH-MN520198-2019	2019	Anhui, CHN	MN520198	GXBS03-2022	2022	Baise, GX, CHN	OR659158
CHHLJ-MZ802955-2020	2020	Heilongjiang, CHN	MZ802955	GXBS04-2022	2022	Baise, GX, CHN	OR659159
CHSD-OM256446-2021	2021	Shandong, CHN	OM256446	GXBS05-2022	2022	Baise, GX, CHN	OR659160
CHGX-OQ547740-2021	2021	Guangxi, CHN	OQ547740	GXBS06-2022	2022	Baise, GX, CHN	OR659161
CHGX-OQ736717-2021	2021	Guangxi, CHN	OQ736717	GXBS07-2022	2022	Baise, GX, CHN	OR659162
CHGX-OQ736716-2022	2022	Guangxi, CHN	OQ736716	GXBS08-2022	2022	Baise, GX, CHN	OR659163
CHHuN-OR269935-2023	2023	Hunan, CHN	OR269935	GXBS09-2022	2022	Baise, GX, CHN	OR659164
Lao-KX118527-2016	2016	Lao	KX118527	GXBS10-2022	2022	Baise, GX, CHN	OR659165
				GXBS11-2022	2022	Baise, GX, CHN	OR659166
				GXBS12-2022	2022	Baise, GX, CHN	OR659167
				GXBS13-2022	2022	Baise, GX, CHN	OR659168
				GXBS14-2022	2022	Baise, GX, CHN	OR659169
				GXGG01-2022	2022	Guigang, GX, CHN	OR659174
				GXGG02-2022	2022	Guigang, GX, CHN	OR659175
				GXGG03-2022	2022	Guigang, GX, CHN	OR659176
				GXGG04-2022	2022	Guigang, GX, CHN	OR659177
				GXGG05-2022	2022	Guigang, GX, CHN	OR659178
				GXGG06-2022	2022	Guigang, GX, CHN	OR659179
				GXGG07-2022	2022	Guigang, GX, CHN	OR659180
				GXYL01-2023	2023	Yulin, GX, CHN	OR659184

Supplementary Table S3. The information of N gene of PDCoV strains in this study.

Virus strain	Date	Origin	Accession No.	Virus strain	Date	Origin	Accession No.

CHAH-KP757890-2004	2004	Anhui, CHN	KP757890	USA-KR265847-2014	2014	USA	KR265847
CHHK-JQ065042-2009	2009	Hong Kong, CHN	JQ065042	USA-KR265848-2014	2014	USA	KR265848
CHHK-JQ065043-2010	2010	Hong Kong, CHN	JQ065043	USA-KR265849-2014	2014	USA	KR265849
CHHK-NC039208-2010	2010	Hong Kong, CHN	NC039208	USA-KR265850-2014	2014	USA	KR265850
CHSD-MF431743-2014	2014	Shandong, CHN	MF431743	USA-KR265851-2014	2014	USA	KR265851
CHHB-KP757891-2014	2014	Hebei, CHN	KP757891	USA-KR265852-2014	2014	USA	KR265852
CHHN-KT336560-2014	2014	Hennan, CHN	KT336560	USA-KR265853-2014	2014	USA	KR265853
CHJS-KU665558-2014	2014	Jiangsu, CHN	KU665558	USA-KR265854-2014	2014	USA	KR265854
CHHuN-KY513724-2014	2014	Hunan, CHN	KY513724	USA-KR265855-2014	2014	USA	KR265855
CHSD-MF431742-2015	2015	Shandong, CHN	MF431742	USA-KR265856-2014	2014	USA	KR265856
CHHeN-MN942260-2015	2015	Henan, CHN	MN942260	USA-KR265857-2014	2014	USA	KR265857
CHJX-KR131621-2015	2015	Jiangxi, CHN	KR131621	USA-KR265858-2014	2014	USA	KR265858
CHNH-KU981059-2015	2015	Heilongjiang, CHN	KU981059	USA-KR265859-2014	2014	USA	KR265859
CHShaanxi-KT021234-2015	2015	Shaanxi, CHN	KT021234	USA-KR265860-2014	2014	USA	KR265860
CHTW-MW854634-2015	2015	Taiwan, CHN	MW854634	USA-KR265861-2014	2014	USA	KR265861
CHGX-KU204697-2015	2015	Guangxi, CHN	KU204697	USA-KR265862-2014	2014	USA	KR265862
CHGS-MF642322-2016	2016	Gansu, CHN	MF642322	USA-KR265863-2014	2014	USA	KR265863
CHGS-MF642323-2016	2016	Gansu, CHN	MF642323	USA-KR265864-2014	2014	USA	KR265864
CHSC-MK355396-2016	2016	Sichuan, CHN	MK355396	USA-KR265865-2014	2014	USA	KR265865
CHHB-KY129986-2016	2016	Hubei, CHN	KY129986	USA-KJ567050-2014	2014	USA	KJ567050
CH-KX443143-2016	2016	Henan, CHN	KX443143	USA-KJ569769-2014	2014	USA	KJ569769
CHHN-MG832584-2016	2016	Henan, CHN	MG832584	USA-KJ584355-2014	2014	USA	KJ584355
CHGD-KY363867-2016	2016	Guangdong, CHN	KY363867	USA-KJ584356-2014	2014	USA	KJ584356
CHGD-KY363868-2016	2016	Guangdong, CHN	KY363868	USA-KJ584357-2014	2014	USA	KJ584357
CHGD-MF280390-2016	2016	Guangdong, CHN	MF280390	USA-KJ584358-2014	2014	USA	KJ584358
CHGD-MH715491-2016	2016	Guangdong, CHN	MH715491	USA-KJ584359-2014	2014	USA	KJ584359
CHJX-KY293677-2016	2016	Jiangxi, CHN	KY293677	USA-MZ291567-2014	2014	USA	MZ291567

CHJX-KY293678-2016	2016	Jiangxi, CHN	KY293678	USA-KJ481931-2014	2014	USA	KJ481931
CHJS-KY513725-2016	2016	Jiangsu, CHN	KY513725	USA-KJ620016-2014	2014	USA	KJ620016
GHSH-MF041982-2016	2016	Shanghai, CHN	MF041982	USA-KT381613-2014	2014	USA	KT381613
CHCQ-MZ772936-2016	2016	Chongqing, CHN	MZ772936	USA-KM012168-2014	2014	USA	KM012168
CHTJ-KY065120-2016	2016	Tianjin, CHN	KY065120	USA-KX022602-2015	2015	USA	KX022602
CHGX-MZ388469-2016	2016	Guangxi, CHN	MZ388469	USA-KX022603-2015	2015	USA	KX022603
CHGS-MF682324-2017	2017	Gansu, CHN	MF682324	USA-KX022604-2015	2015	USA	KX022604
CHGS-MF642325-2017	2017	Gansu, CHN	MF642325	USA-KX022605-2015	2015	USA	KX022605
CHSC-MK572803-2017	2017	Sichuan, CHN	MK572803	USA-KR150443-2015	2015	USA	KR150443
CHSC-MK330604-2017	2017	Sichuan, CHN	MK330604	JPN-LC260038-2014	2014	JPN	LC260038
CHSC-MK211169-2017	2017	Sichuan, CHN	MK211169	JPN-LC260039-2014	2014	JPN	LC260039
CHHB-MF948005-2017	2017	Hubei, CHN	MF948005	JPN-LC260040-2014	2014	JPN	LC260040
CHHeB-MG242062-2017	2017	Hebei, CHN	MG242062	JPN-LC260041-2014	2014	JPN	LC260041
CHJS-MN249445-2017	2017	Jiangsu, CHN	MN249445	JPN-LC260042-2014	2014	JPN	LC260042
CHHG-MF095123-2017	2017	Hubei, CHN	MF095123	JPN-LC260043-2014	2014	JPN	LC260043
CHGX-MZ388470-2017	2017	Guangxi, CHN	MZ388470	JPN-LC260044-2014	2014	JPN	LC260044
CHGX-MZ388471-2017	2017	Guangxi, CHN	MZ388471	JPN-LC260045-2016	2016	JPN	LC260045
CHGX-MZ388472-2017	2017	Guangxi, CHN	MZ388472	KOR-KM820765-2014	2014	KOR	KM820765
CHGX-MT524762-2017	2017	Guangxi, CHN	MT524762	KOR-KY354363-2016	2016	KOR	KY354363
CHGX-MT524763-2017	2017	Guangxi, CHN	MT524763	KOR-KY354364-2016	2016	KOR	KY354364
CHGX-MT524764-2017	2017	Guangxi, CHN	MT524764	KOR-KY354365-2014	2014	KOR	KY354365
CHGX-MT524765-2017	2017	Guangxi, CHN	MT524765	KOR-MG837130-2016	2016	KOR	MG837130
CHGX-MT524766-2017	2017	Guangxi, CHN	MT524766	KOR-MG837131-2016	2016	KOR	MG837131
CHGX-MN025260-2017	2017	Guangxi, CHN	MN025260	KOR-MG837132-2016	2016	KOR	MG837132
CHSC-MK330605-2018	2018	Sichuan, CHN	MK330605	KOR-MG837133-2016	2016	KOR	MG837133
CHSC-MK005882-2018	2018	Sichuan, CHN	MK005882	KOR-KY926512-2016	2016	KOR	KY926512
CHSC-KT266822-2018	2018	Sichuan, CHN	KT266822	Thailand-KX361343-2013	2013	Thailand	KX361343

CHHN-MH708123-2018	2018	Henan, CHN	MH708123	Thailand-KX361344-2013	2013	Thailand	KX361344
CHHN-MH708124-2018	2018	Henan, CHN	MH708124	Thailand-KU984334-2015	2015	Thailand	KU984334
CHHN-MH708125-2018	2018	Henan, CHN	MH708125	Thailand-KU051641-2015	2015	Thailand	KU051641
CHJX-KY625638-2018	2018	Jiangxi, CHN	KY625638	Thailand-KX361345-2015	2015	Thailand	KX361345
CHJX-KY625639-2018	2018	Jiangxi, CHN	KY625639	Thailand-KU051649-2015	2015	Thailand	KU051649
CHJX-KY625640-2018	2018	Jiangxi, CHN	KY625640	Thailand-MZ802772-2016	2016	Thailand	MZ802772
CHJX-MK625641-2018	2018	Jiangxi, CHN	MK625641	Thailand-MZ802773-2016	2016	Thailand	MZ802773
CHGX-MZ388473-2018	2018	Guangxi, CHN	MZ388473	Thailand-MZ802774-2016	2016	Thailand	MZ802774
CHGX-MZ388474-2018	2018	Guangxi, CHN	MZ388474	Thailand-MZ802775-2016	2016	Thailand	MZ802775
CHGX-MK359104-2018	2018	Guangxi, CHN	MK359104	Thailand-MZ802777-2016	2016	Thailand	MZ802777
CHGX-MN173782-2018	2018	Guangxi, CHN	MN173782	Vietnam-KX834351-2015	2015	Vietnam	KX834351
CHGX-MN173779-2018	2018	Guangxi, CHN	MN173779	Vietnam-KX834352-2015	2015	Vietnam	KX834352
CHGX-MN173780-2018	2018	Guangxi, CHN	MN173780	Vietnam-MH700628-2015	2015	Vietnam	MH700628
CHGX-MN173781-2018	2018	Guangxi, CHN	MN173781	Vietnam-MH118332-2015	2015	Vietnam	MH118332
CHGX-MT648377-2018	2018	Guangxi, CHN	MT648377	Vietnam-MZ802776-2016	2016	Vietnam	MZ802776
CHGX-MT648378-2018	2018	Guangxi, CHN	MT648378	Lao-KX118627-2016	2016	Lao	KX118627
CHGX-MT648379-2018	2018	Guangxi, CHN	MT648379	GXBS01-2020	2020	Baise, GX, CHN	OR659185
CHGX-MT648380-2018	2018	Guangxi, CHN	MT648380	GXBS02-2020	2020	Baise, GX, CHN	OR659186
CHGX-MT648381-2018	2018	Guangxi, CHN	MT648381	GXBS03-2020	2020	Baise, GX, CHN	OR659187
CHGX-MT648382-2018	2018	Guangxi, CHN	MT648382	GXBS01-2021	2021	Baise, GX, CHN	OR659188
CHGX-MN520203-2018	2018	Guangxi, CHN	MN520203	GXBS02-2021	2021	Baise, GX, CHN	OR659189
CHGX-MN058073-2018	2018	Guangxi, CHN	MN058073	GXBS01-2022	2022	Baise, GX, CHN	OR659190
CHSC-MK993519-2019	2019	Sichuan, CHN	MK993519	GXBS02-2022	2022	Baise, GX, CHN	OR659191
CHSD-MN520191-2019	2019	Shandong, CHN	MN520191	GXBS03-2022	2022	Baise, GX, CHN	OR659192
CHTJ-MT663769-2019	2019	Tianjin, CHN	MT663769	GXBS04-2022	2022	Baise, GX, CHN	OR659193
CHGX-MT648383-2019	2019	Guangxi, CHN	MT648383	GXBS05-2022	2022	Baise, GX, CHN	OR659194
CHGX-MT648384-2019	2019	Guangxi, CHN	MT648384	GXBS06-2022	2022	Baise, GX, CHN	OR659195

CHGX-MT648385-2019	2019	Guangxi, CHN	MT648385	GXBS07-2022	2022	Baise, GX, CHN	OR659196
CHGX-MT648386-2019	2019	Guangxi, CHN	MT648386	GXBS08-2022	2022	Baise, GX, CHN	OR659197
CHGX-MT648387-2019	2019	Guangxi, CHN	MT648387	GXBS09-2022	2022	Baise, GX, CHN	OR659198
CHHLJ-MZ802955-2020	2020	Heilongjiang, CHN	MZ802955	GXBS10-2022	2022	Baise, GX, CHN	OR659199
CHSD-OM256446-2021	2021	Shandong, CHN	OM256446	GXBS11-2022	2022	Baise, GX, CHN	OR659200
CHGX-OQ547740-2021	2021	Guangxi, CHN	OQ547740	GXBS12-2022	2022	Baise, GX, CHN	OR659201
CHGX-OQ736717-2021	2021	Guangxi, CHN	OQ736717	GXBS13-2022	2022	Baise, GX, CHN	OR659202
CHGX-OQ736716-2022	2022	Guangxi, CHN	OQ736716	GXBS14-2022	2022	Baise, GX, CHN	OR659203
CHHuN-OR269935-2023	2023	Hunan, CHN	OR269935	GXCZ01-2021	2021	Chongzuo, GX, CHN	OR659204
				GXCZ02-2021	2021	Chongzuo, GX, CHN	OR659205
				GXCZ03-2021	2021	Chongzuo, GX, CHN	OR659206
				GXCZ04-2021	2021	Chongzuo, GX, CHN	OR659207
				GXGG01-2022	2022	Guigang, GX, CHN	OR659208
				GXGG02-2022	2022	Guigang, GX, CHN	OR659209
				GXGG03-2022	2022	Guigang, GX, CHN	OR659210
				GXGG04-2022	2022	Guigang, GX, CHN	OR659211
				GXGG05-2022	2022	Guigang, GX, CHN	OR659212
				GXGG06-2022	2022	Guigang, GX, CHN	OR659213
				GXGG07-2022	2022	Guigang, GX, CHN	OR659214
				GXNN01-2021	2021	Nanning, GX, CHN	OR659215
				GXNN02-2021	2021	Nanning, GX, CHN	OR659216
				GXNN03-2021	2021	Nanning, GX, CHN	OR659217
				GXYL01-2023	2023	Yulin, GX, CHN	OR659218
